# Tourette Syndrome: Complementary Insights from Measures of Cognitive Control, Eyeblink Rate, and Pupil Diameter

**DOI:** 10.3389/fpsyt.2015.00095

**Published:** 2015-06-29

**Authors:** Jordan A. Tharp, Carter Wendelken, Carol A. Mathews, Elysa J. Marco, Herbert Schreier, Silvia A. Bunge

**Affiliations:** ^1^Department of Psychology, University of California Berkeley, Berkeley, CA, USA; ^2^Helen Wills Neuroscience Institute, University of California Berkeley, Berkeley, CA, USA; ^3^Department of Psychiatry, University of California San Francisco School of Medicine, San Francisco, CA, USA; ^4^Department of Neurology, University of California San Francisco School of Medicine, San Francisco, CA, USA; ^5^Department of Psychiatry, UCSF Benioff Children’s Hospital Oakland, Oakland, CA, USA

**Keywords:** Tourette syndrome, eye-blink rate, pupillometry, cognitive control, executive functions, dopamine, norepinephrine, anxiety

## Abstract

Some individuals with Tourette syndrome (TS) have severe motoric and vocal tics that interfere with all aspects of their lives, while others have mild tics that pose few problems. We hypothesize that observed tic severity reflects a combination of factors, including the degree to which dopaminergic (DA) and/or noradrenergic (NE) neurotransmitter systems have been affected by the disorder, and the degree to which the child can exert cognitive control to suppress unwanted tics. To explore these hypotheses, we collected behavioral and eyetracking data from 26 patients with TS and 26 controls between ages 7 and 14, both at rest and while they performed a test of cognitive control. To our knowledge, this is the first study to use eyetracking measures in patients with TS. We measured spontaneous eyeblink rate as well as pupil diameter, which have been linked, respectively, to DA and NE levels in the central nervous system. Here, we report a number of key findings that held when we restricted analyses to unmedicated patients. First, patients’ accuracy on our test of cognitive control accounted for fully 50% of the variance in parentally reported tic severity. Second, patients exhibited elevated spontaneous eyeblink rates compared to controls, both during task performance and at rest, consistent with heightened DA transmission. Third, although neither task-evoked pupil dilation nor resting pupil diameter differed between TS patients and controls, pupil diameter was positively related to parentally reported anxiety levels in patients, suggesting heightened NE transmission in patients with comorbid anxiety. Thus, with the behavioral and eyetracking data gathered from a single task, we can gather objective data that are related both to tic severity and anxiety levels in pediatric patients with TS, and that likely reflect patients’ underlying neurochemical disturbances.

## Introduction

Tourette Syndrome (TS) is a neurodevelopmental disorder characterized by non-purposeful, rapid, recurrent vocalizations, and movements, frequently accompanied by unusual and uncomfortable sensations or urges (1–3). Some individuals with TS have severe tics that interfere with all aspects of their lives, whereas others have only mild tics that pose few problems. Further, a diagnosis of “pure” TS is the exception rather than the rule; over 80% of TS patients have been diagnosed with comorbid mental health conditions, most frequently attention-deficit hyperactivity disorder (ADHD) and obsessive–compulsive disorder (OCD) (4–6). Thus, a better understanding of individual differences among patients with TS could lead to evidence-based, individualized treatment plans.

Tourette syndrome, ADHD, and OCD have all been characterized as disorders implicating frontostriatal circuitry (7–10), which underpins both motoric and cognitive control (11). While altered motor control is a defining characteristic of TS, there is mixed evidence of altered cognitive control in this disorder (11). Here, we tested whether behavioral measures of cognitive control, along with spontaneous eyeblink rate and/or pupil diameter measured at rest and during execution of a cognitive control task, could help to differentiate patients with TS from one another and/or from controls.

Elevated dopamine (DA) levels in the brain have long been thought to be central to the etiology of TS (7, 12, 13). However, the medications that have proven most effective in the treatment of TS broadly influence monoamine function, affecting DA, norepinephrine (NE), and/or serotonin, or elevate GABA levels in the brain (14). Thus, the pathophysiology of TS may involve multiple neurotransmitter systems and/or may differ across patient subtypes. Notably, prefrontal cortex, a locus of cognitive control, is especially sensitive to levels of NE and DA. In our prior fMRI research, we observed greater prefrontal activation in TS patients relative to age-matched controls during engagement of cognitive control (11). Here, we explore whether ocular measures that have been linked to DA and NE function could be used to better characterize the neurochemical disturbances exhibited by individual patients.

Dopaminergic activity in the brain, particularly D1 receptor activity, has been linked to spontaneous eye-blink rate (15–17). Prior studies have reported higher blink rates or blink reflexes in TS patients compared to controls (18, 19). However, eyelid movements are a common tic (20), and it is unclear whether previous findings simply reflect eyelid movement tics or whether even regular eyeblinks occur more frequently in TS, which would be consistent with elevated tonic DA transmission in the brain. Here, we sought to harness the temporal precision of eyetracking technology to separate rapid spontaneous eye blinks from longer-duration events that may reflect the expression of ocular tics. This approach has two advantages over video coding of blinks: less laborious and more precise. Using this measure, we tested for differences in spontaneous blink rate between TS patients and controls, and tested for correlations with symptom severity.

NE release from the locus coeruleus (LC) leads to pupil dilation as well as heightened arousal; thus, pupil diameter is an indirect indicator of NE activity in the brain (21, 22). We sought to test for altered pupil diameter in TS patients at rest and/or during performance of a challenging task, which would indirectly suggest, respectively, altered tonic and/or phasic NE release. We also tested for relationships between pupil diameter and symptom severity in patients.

## Materials and Methods

All procedures were approved by the University of California at Berkeley Institutional Review Board.

### Participants

#### Patients

Children diagnosed with TS were referred to the study by an advertisement placed with the Tourette Syndrome Association local chapter, pediatric neurologists and psychiatrists at the Departments of Neurology and Psychiatry at UCSF, re-contact of participants in the San Francisco Bay Area who participated in a genetic study of TS and noted that they were interested in participating in additional TS-related research, and psychiatrists at Children’s Hospital of Oakland. Children between the ages 7 and 14 and with a primary diagnosis of TS were eligible. Children with common comorbidities of OCD, ADHD, mood, and/or anxiety disorders were included, but all other psychological disorders (psychosis, Autism spectrum, etc.) were excluded. Prematurely born children (less than 37 weeks gestation), children with congenital or acquired neurological disorders, and children with a history of serious head or eye trauma were also ineligible for participation in this study.

#### Controls

A group of children matched to the patients for age, gender, and socioeconomic status was recruited from the San Francisco Bay Area through advertisements placed in local parent magazines and online forums. Similar to the patients, children born prematurely, with psychiatric disorders, congenital or acquired neurological disorders, or history of serious head or eye trauma were excluded.

#### Procedure

Interested parents were contacted by phone and asked eligibility screening questions about their children by members of the research team. Parents who signed up for the study were asked to have their child withhold from any allergy or cold medications on the day of testing and to ensure that their child did not wear contact lenses. Upon arrival at the testing suite, parents signed consent forms on behalf of their children, and children aged 12–14 signed an assent form.

Sixty children (age range, 7–14 years, mean = 10.24 years) started the study, of whom 28 had a TS diagnosis and 32 were healthy age-matched controls. Of these, six controls and two patients were excluded: one control because they took allergy medication on the morning of testing, and the other seven participants due to technical difficulties resulting in loss of data. Of the remaining 52 children (26 patients and 26 controls), 7 (5 patients and 2 controls) were excluded from analysis due to low-quality eye-tracking data (<35% valid), and 5 (2 patients and 3 controls) were excluded from analysis due to poor behavioral performance (<55% total accuracy). Thus, 19 patients (mean age = 10.89, 9 females) and 21 controls (mean age = 11.04, 11 females) were included in our analyses.

Three controls and four patients wore glasses during testing. Parents were asked to provide the type and dosage of their child’s current medications and supplements. Eight patients were currently taking medications for the treatment of TS, ADHD, OCD, and/or anxiety. Within the TS group, 14 children were TS-only (2 were medicated), 7 children were TS with comorbid ADHD (2 with additional anxiety and 1 medicated), 5 were TS with comorbid ADHD and OCD (4 were medicated), and 2 were TS with comorbid OCD (1 with additional anxiety and medicated). Of the 19 patients with usable data, 13 were unmedicated. As reported below, the key findings were obtained regardless of whether medicated patients were included or excluded from analyses.

### Standardized assessments

All participants completed the Wechsler Abbreviated Scale of Intelligence (WASI), which comprises four subtests: Vocabulary, Similarities, Block Design, and Matrix Reasoning, as well as the Forward Digit Span task from the Wechsler Intelligence Scale for Children. The following clinical measures were administered to parents and/or their children: Child Behavior Checklist (CBCL) (23), Behavior Rating Inventory of Executive Function (BRIEF) (24), Multidimensional Anxiety Scale for Children (MASC) (25), Yale Global Tic Severity Scale (YGTSS) (26), Conners’ ADHD Parent Rating Scale (27), and Children’s Yale-Brown Obsessive–Compulsive Scale (CY-BOCS) (28). To ensure that our patients and controls did not differ on socioeconomic variables, we asked parents to complete the MacArthur scale of subjective social status (29). Additionally, to ensure that participants did not differ in terms of maturation level, birth dates and stage of pubertal development using the Pubertal Development Scale were obtained (30). All assessments were conducted by the first author, a trained research assistant.

Patients were referred to the study based on a clinical diagnosis of TS, and we used the YGTSS as an index of tic severity. This semi-structured interview, administered to the patient and parent(s) together, measures the severity of both motor and phonic tics experienced during the previous week. Interviewer observations were also recorded. For more information about the YGTSS and other clinical assessments, please see Supplementary Material.

### Eyetracking data collection

#### Data Acquisition

Children began the testing session seated in front of a Tobii T120 eyetracker (17″ monitor, 1280 × 1024 pixel resolution) at a distance of 50–80 cm from the monitor. Stimuli were presented using a synchronized computer with Tobii E-prime Software Extensions (Psychology Software Tools, Pittsburgh, PA, USA). Data were collected in a windowless room with constant fixed overhead light fixtures to minimize luminance changes.

Data were captured at a temporal resolution of 120 Hz from Tobii built-in camera, yielding data for left and right eyes separately every 8.3 ms. Each individual was calibrated at the start of the session. Next, participants focused on a fixation cross (+) in the center of the screen for 30 s. During this time, the participants were asked to focus on the screen without moving around, looking away, or closing their eyes. Blinking and looking around the screen were allowed, and patients were informed that they could tic if needed.

#### Cognitive Control Task

**Figure 1:**
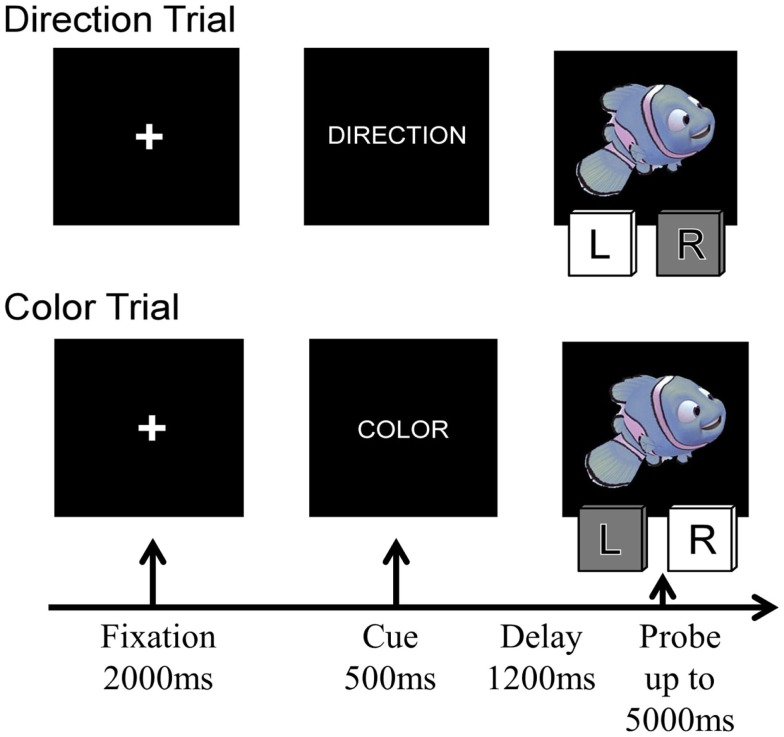
**Four sample trials of the Nemo task, showing the sequence of stimuli presented on each trial**. The condition labels and the indicated correct responses correspond to the rule “Blue is left, red is right.”

Children then performed the “Nemo task” [modified from Baym et al. (11)] while eyetracking data were collected (Figure 1). This task, described in detail below, requires participants to switch flexibly from one task rule to another. The task involves three distinct manipulations: (1) rule type: a manipulation of rule representation, comparing arbitrary with non-arbitrary stimulus-response mappings, (2) switching: whether the rule switches or repeats, and (3) congruency: whether a stimulus would elicit the same response or a different response depending on whether participants are required to make a judgment based on the stimulus color or orientation. On congruent trials, participants would be correct regardless of which rule they followed. On incongruent trials, however, only the currently relevant task rule specifies the correct response. Thus, performance on incongruent trials serves as a litmus test of the ability to switch flexibly between two task rules, selecting the appropriate response while ignoring the currently irrelevant stimulus dimension.

On every trial, children were presented with a cue stimulus indicating the currently relevant rule – the word “COLOR” or “DIRECTION” and were then presented with a cartoon stimulus – a red or blue fish facing either left or right. On Direction trials, they had to press the leftward of two computer keys (the “F” key) when the cartoon faced left and the rightward key (the “J” key) when the stimuli faced right. On Color trials, they were asked to press the “F” or “J” key depending on the color of the cartoon stimulus. Red and blue stimuli were pseudo-randomly assigned to the “F” or “J” key for each participant. On Congruent trials, the two stimulus features were associated with the same response to the stimulus, whereas on incongruent trials, the color of the stimulus indicated the opposite response from the direction the cartoon was facing. On repetition trials, the rule was the same as on the immediately preceding trial (i.e., Direction preceded by Direction or Color preceded by Color), whereas on Switch trials, the rule changed (i.e., Direction preceded by Color or Color preceded by Direction).

Each trial began with a 2000 ms fixation cross, followed by the rule (or cue) for 500 ms, a 1200 ms delay screen, and then the probe (cartoon stimulus) was presented. Participants were given up to 5000 ms to make their response (see Figure 1); the task continued without feedback. Children received two practice blocks prior to beginning the task: one block per rule, with 12 trials each (24 practice trials total). Neutral stimuli were used during the practice trials to assist with learning which key to press for the cues (a gray arrow facing the left or right for direction cues and red and blue squares for color cues). Between each block, children were given a break and could proceed to the next block when they were ready.

After finishing the practice trials, participants completed six blocks of 16 trials each, for a total of 96 trials. Four blocks were single-rule blocks (two each of Direction and Color) and two blocks were Mixed-rule blocks, in which Color and Direction trials were intermixed pseudo-randomly. Across the two mixed-rule blocks, participants completed equal numbers of Color and Direction trials, Switch and Repeat trials, and Congruent and Incongruent trials. Task blocks were presented for all participants in the following order: Direction, Color, Mixed, Mixed, Color, and Direction. Nine controls and one patient completed five 16-trial Mixed-rule blocks, with no single-rule blocks.

#### Eyetracking Measures of Interest

Pupil diameter (in millimeter) was collected separately for each eye every 8.3 ms. Since pupil diameter was highly correlated between the left and right eyes, we computed average pupil diameter across the two eyes. We performed linear interpolation across gaps in the pupil data and then extracted the pupil diameter timecourse for each Nemo task trial. Next, these trial timecourses were averaged across trials to produce pupil diameter timecourses associated with selected task conditions. To produce a summary measure of task-based pupil diameter, we averaged data from the first 3 s of each trial, which included the appearance of the rule cue, delay, probe stimulus, and time for response. For a measure of resting pupil diameter, we calculated the mean pupil diameter over the 30-s baseline period collected prior to the Nemo task.

The two principal causes of missing data are eye blinks and head movements that cause the eyes to move outside of the eyetracker field of view. Before comparing blink rates between patients and controls, we sought to ascertain the nature of gaps in the eyetracking data, including blinks, tics, and other events. We plotted a histogram of gaps in the eyetracking data, binned by duration in 100 ms intervals, excluding gaps below 100 ms. Gaps of between 100 and 500 ms were considered to be valid blinks [cf. (31, 32)], and blink rate was calculated as the number of valid blinks divided by the total valid duration, where valid duration was the total duration minus the duration of longer (>500 ms) gaps. We calculated blink rate in the same manner for the 30-s baseline period.

## Results

### Questionnaire measures

Demographic variables and standardized assessment data are presented in Table 1 for controls, all patients, and unmedicated patients. Significant group differences were observed regardless of which set of patients was considered for full-scale IQ [Wechsler Abbreviated Scale of Intelligence (WASI)], behavioral self-regulation (BRIEF), child behavior problems (CBCL), ADHD symptoms (Conner’s), obsessive–compulsive symptoms (CY-BOCS), and tic severity (YGTSS). In addition, patients exhibited a trend toward higher anxiety levels (MASC Anxiety Index) than controls. Gender, age, and pubertal development status were well matched across groups. In general, unmedicated patients were reported to have milder symptoms than medicated patients, likely reflecting the fact that more severely affected patients are more likely to seek and receive prescription medications.

**Table 1:** **Group differences in demographics and questionnaires, considering all TS patients as well as unmedicated TS patients only**.

	Control	All TS	*t*, *p* (All)	Unmed TS	*t*, *p* (Unmed)
				
	Mean (SD)	Mean (SD)		Mean (SD)	
*N* (total)	26	26	n/a	18	n/a
*N* (analyzed)	21	19	n/a	13	n/a
% Male	54.4	61.5	1.4, 17	52.6	
Age	10.5 (2)	10.4 (2)		10.1 (2)	
Pubertal development	11.9 (4)	12.3 (4)		12.8 (4)	
SES	7.6 (1)	7.2 (2)	1.0, 0.35	6.9 (2)	1.3, 0.19
Digit span score	10.5 (2)	9.3	1.7, 0.11	9.6 (2)	1.1, 0.27
**WASI**	121.1 (14)	112.5 (13)	**2.3, 0.03**	112.0 (13)	**2.2, 0.04**
**BRIEF**	49.6 (10)	60.2 (12)	**−3.5, 0.001**	59.1 (13)	**−2.8, 0.01**
**CBCL**	48.8 (13)	59.2 (11)	**−3.1, <0.005**	58.2 (12)	**−2.5, 0.01**
**Conners’ ADHD**	51.1 (8)	60.7 (14)	**−3.1, <0.005**	61.6 (16)	**−2.7, 0.01**
**CY-BOCS**	0	6.3 (8)	**−3.9, <0.001**	4.7 (8)	**−3.1, 0.02**
**YGTSS**	0	21.2 (13)	**−8.1, <0.001**	17.7 (7)	**−10.9, 0.001**
MASC	64.4 (18)	73.1 (14)	−1.7, 0.11	74.6 (11)	−1.9, 0.06

*SES, Socioeconomic Status; WASI, Wechsler Abbreviated Scale of Intelligence; BRIEF, Behavior Rating Inventory of Executive Function (global measure); CBCL, Child Behavior Checklist; Conners’ ADHD, Conners’ ADHD Index; CY-BOCS, Children’s Yale–Brown Obsessive–Compulsive Scale; YGTSS, Yale Global Tic Severity Scale; MASC, Multidimensional Anxiety Scale for Children; n/a, not applicable. T-tests are reported for all TS patients vs. controls, as well as for unmedicated TS patients vs. controls. Significant group differences are featured in bolded text. Statistics are not reported for t-values <1*.

### Nemo task performance

The majority of patients and controls understood the Nemo task instructions and performed well above chance. However, following preliminary behavioral screening, three controls (ages 8, 9, and 10 years) and two patients (ages 10 and 12 years) were excluded from subsequent analyses because their overall accuracy levels were close to chance (≤55% correct).

To test for effects of block type on performance, we ran Group × Block (Direction, Color, Mixed) ANOVAs for incongruent trial accuracy and response times (RTs). Effects of block type on behavior and ocular measures are shown in Figures S1 and S2 in Supplementary Material. There was a main effect of block type on accuracy, with Direction > Mixed > Color (*p*’s < 0.001). There was also a marginal effect of Group on accuracy, such that patients demonstrated reduced accuracy compared to controls (*p* = 0.07). This group difference was particularly apparent for Mixed blocks (*p* = 0.05), though the Block × Group interaction was not significant (*p* = 0.19). There was also a main effect of Block type on RT (*p* < 0.001). Both patients and controls demonstrated increased RTs for Mixed blocks, relative to both Color and Direction blocks, and for Color blocks relative to Direction blocks (*p*’s < 0.05). There was no main effect of Group or Block × Group interaction for RTs (*p*’s > 0.2). Notably, block type had differing effects on accuracy and RT: whereas accuracy was lowest on Color blocks, RTs were slowest for Mixed blocks. This apparent speed-accuracy tradeoff suggests that participants were more careful when required to switch between rules.

**Figure 2:**
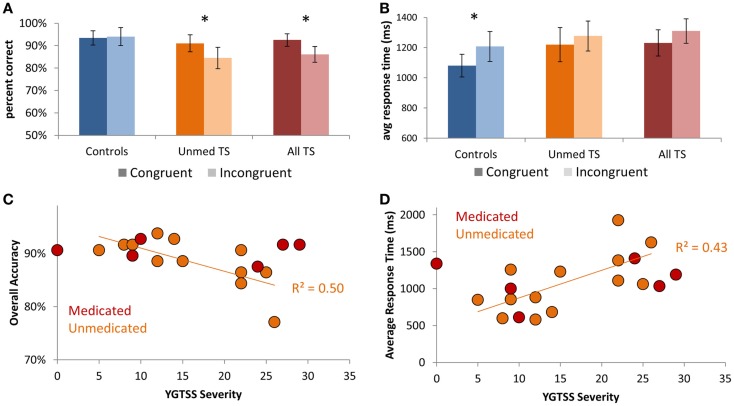
**Performance on Mixed blocks of the Nemo task**. **(A)** Patients exhibited a larger decrement in performance on Incongruent vs. Congruent trials than did controls. **(B)** Controls demonstrated increased response times for Incongruent relative to Congruent trials. **(C)** Scatter-plot showing the relationship between tic severity (YGTSS severity index) and accuracy in unmedicated TS patients. **(D)** Scatter-plot showing the relationship between tic severity and average response times in unmedicated TS patients. Error bars indicate within-subjects standard error. Asterisks denote *p* < 0.05.

Focusing on the Mixed-rule blocks, for which there was a group difference in accuracy, we ran separate Group × Condition ANOVAs for each manipulation: Congruency, Switching, and Rule type. For accuracy, we observed a main effect of Congruency [Congruent > Incongruent; *F*(1,38) = 6.45, *p* = 0.02] that was qualified by a Group × Congruency interaction [*F*(1,38) = 9.38, *p* = 0.004], such that TS patients but not controls demonstrated decreased accuracy on Incongruent relative to Congruent trials (Figure 2A; All TS: *p* = 0.001; Unmed TS: *p* = 0.01; controls: *p* > 0.20). Thus, patients had a greater tendency than controls to apply the incorrect rule when the two rules were associated with opposite responses. We also observed a main effect of Congruency on RTs [Figure 2B; *F*(1,38) = 6.88, *p* = 0.01], with slower RTs for Incongruent than Congruent trials. Although no main effect was observed for Rule type or Switching on accuracy or RTs (*p*s > 0.2), a Group × Switching interaction was observed for accuracy [*F*(1,38) = 4.94, *p* = 0.03], such that controls but not TS patients tended to demonstrate better accuracy on Repeat than Switch trials (All TS: *p* > 0.2; Unmed TS: *p* > 0.2; controls: *p* = 0.06).

Focusing on individual differences in TS symptom severity among unmedicated patients, we investigated whether those with higher tic severity exhibited worse task performance. We observed strong negative correlations between YGTSS tic severity score and overall accuracy (*r* = −0.72, *p* = 0.006; Figure 2C) – i.e., accuracy on our Nemo task accounted for 50% of the variance in parentally reported tic severity on the YGTSS (*r*^2^ = 0.50). We also observed a positive correlation between tic severity and average RTs (*r* = 0.65, *p* = 0.015; Figure 2D), indicating that patients with more severe tics were both less accurate and slower. These relationships held even when controlling for ADHD symptom severity, as measured by Conner’s ADHD index, OCD symptom severity, as measured by CY-BOCS, or anxiety, as measured by the MASC Anxiety Index (all *p*’s < 0.05). Thus, higher tic severity was strongly associated with worse performance on the Nemo task.

### Spontaneous eyeblink rate: Task-evoked and resting state

**Figure 3:**
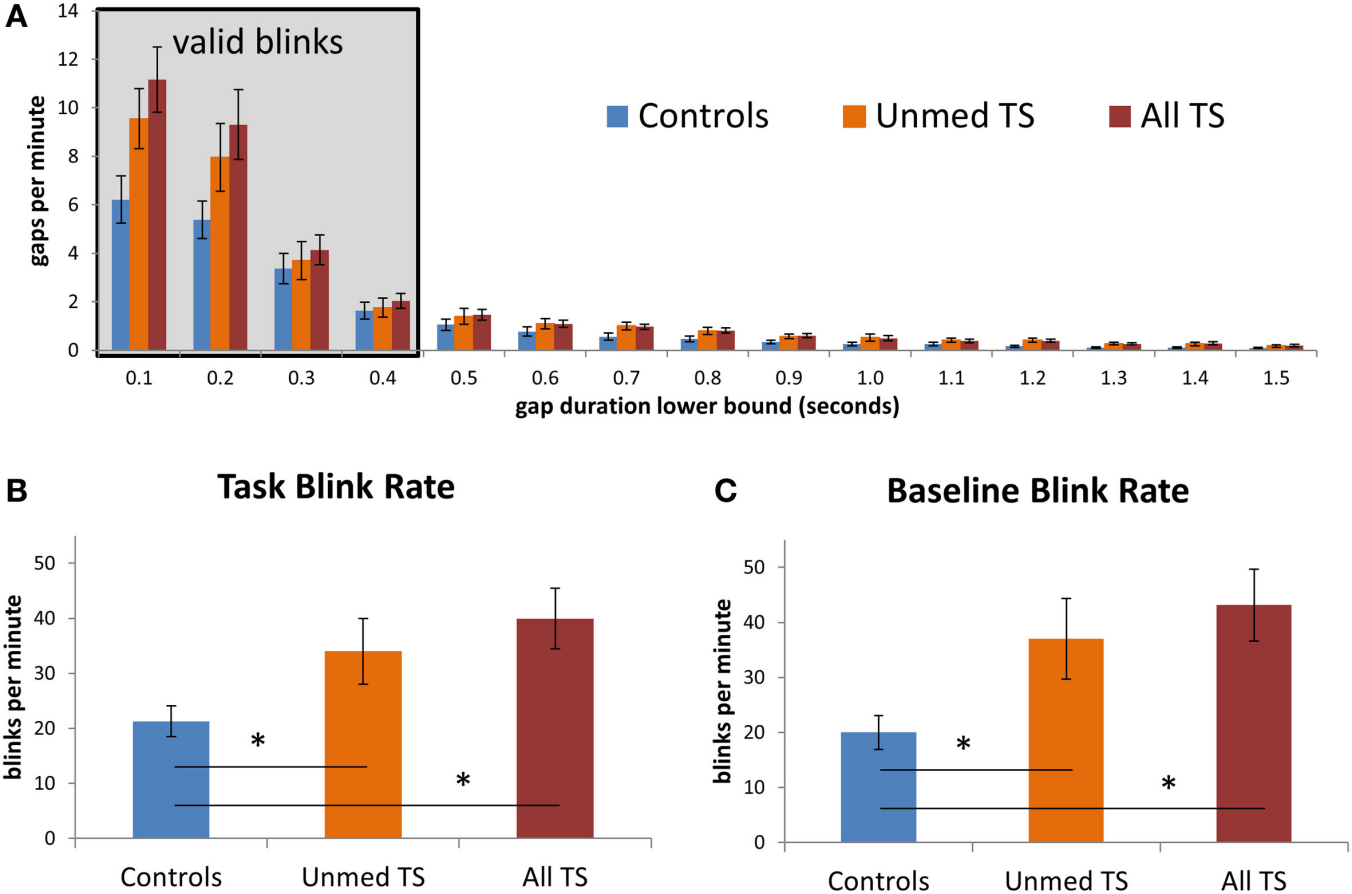
**(A)** Observed rate of gaps in the eyetracking data, at fixed intervals, for patients and controls. Values on the *x*-axis give the lower bound for each interval; the left-most interval includes gaps between 100 and 200 ms in duration, while the rightmost interval includes gaps between 1500 and 1600 ms. The shaded box indicates gaps that were considered to be valid blinks. Gaps of <100 ms were excluded from this graph. **(B)** Average blink rate during task blocks, in controls, unmedicated TS patients, and all TS patients. **(C)** Average blink rate during the 30-s baseline fixation period, for each group. Error bars indicate standard error of the mean. Asterisks denote *p* < 0.05.

We investigated whether the analysis of short-duration gaps in eyetracking data would support prior reports of elevated spontaneous blink rate in TS. In particular, we sought to test whether patients would exhibit a higher rate of short-duration gaps *of the same duration as spontaneous blinks in controls*, leaving aside any longer-duration gaps that could be due to tics. Indeed, the frequency of short-duration gaps (deemed valid blinks; see Figure 3A) is consistent with the finding that patients blinked more frequently than controls [all TS: *t*(38) = −3.01, *p* = 0.005; unmedicated TS: *t*(32) = −2.08, *p* = 0.04; Figure 3B]. Notably, this elevated blink rate among patients could also be detected based on data from the 30-s baseline period [all TS: *t*(38) = −3.21, *p* = 0.003; unmedicated TS: *t*(32) = −2.37, *p* = 0.02; Figure 3C]. Thus, this difference could be observed both when participants performed a task and when they rested and was reliable with as little as 30 s of data.

Given that the TS group had a higher incidence of ADHD than controls (see Table 1) and that altered DA transmission has been reported in ADHD, we next investigated whether the group difference in blink rate would hold when controlling for ADHD severity. Controlling for ADHD severity in an ANOVA, the group difference between All TS patients and controls remained significant [*F*(1,37) = 5.08, *p* = 0.03], and there was a trend-level effect when this analysis was restricted to unmedicated patients vs. controls [*F*(1,31) = 1.99, *p* = 0.17]. Similarly, the difference in blink rate between patients and controls remained significant when controlling for OCD severity [all TS: *F*(1,37) = 8.40, *p* = 0.006; Unmed TS: *F*(1,31) = 4.63, *p* = 0.04]^1^.

Finally, we investigated the relationship between blink rate and reported tic severity in unmedicated patients. Contrary to our expectation of a positive correlation, a trend-level *negative* relationship was observed during task performance (*r* = −0.42, *p* = 0.15). This negative relationship was maintained when controlling separately for ADHD, OCD, and MASC anxiety (correlation *r*-values of −0.56, −0.37, and −0.36, respectively) and was also present in the 30-s baseline period (*r* = −0.36). Thus, although TS patients as a whole blinked more often than controls, higher blink rates did not correspond to higher tic severity. A larger pool of TS patients with and without comorbid conditions is required to clarify the relationship between blink rate and symptom severity across patients, both at rest and during performance of cognitively demanding tasks.

### Pupil diameter: Task-evoked and resting state

**Figure 4:**
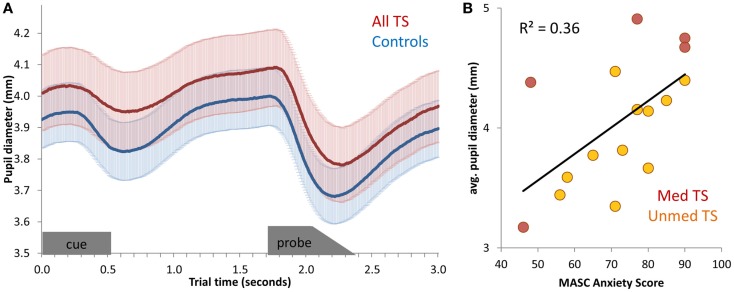
**(A)** Average pupil diameter timecourses, for all TS patients and for controls. Pupil diameter was averaged across all correct mixed-block trials, and then averaged across participants. Error bars (shading) depict standard error of the mean, across participants. The appearance of the cue stimulus and probe stimulus are indicated at the bottom of the graph. **(B)** Scatter-plot of the relationship between anxiety (MASC Anxiety score) and average pupil diameter across unmedicated TS patients.

We first tested for group differences in task-evoked and resting pupil diameter. Task-evoked pupil timecourses demonstrated a characteristic pattern of pupil constriction and dilation in response to task events (Figure 4A). We observed no differences between patients and controls in mean task-evoked pupil diameter or mean resting pupil diameter, either when considering unmedicated patients alone or all TS patients (*p*’s > 0.20). Thus, whereas spontaneous blink rate distinguished between patients and controls, pupil diameter did not.

Next, we tested for correlations between mean pupil diameter during Nemo task performance and symptom severity among patients. No relation between pupil diameter and tic severity or ADHD was observed (all *p*’s > 0.20), and only a marginal positive relationship with OCD was found [all TS: *r*(19) = 0.40, *p* = 0.09; unmedicated TS: *r*(13) = 0.24, *p* > 0.20]. However, there was a positive relationship between pupil diameter during task performance and anxiety, as measured by the MASC [all TS: *r*(16) = 0.60, *p* = 0.02; Unmedicated TS: *r*(11) = 0.65, *p* = 0.03; Figure 4B]. This relationship was also present across all participants [*r*(27) = 0.50, *p* = 0.009], although it was not significant in controls alone [*r*(11) = 0.37, *p* > 0.20]. Thus, larger pupil diameter during task performance was indicative of higher anxiety.

Having found a relationship between MASC scores and pupil diameter during cognitive control task performance, we next tested for a relationship between MASC scores of anxiety and the mean *resting* pupil diameter over the 30-s baseline period obtained prior to Nemo task performance. Again, pupil diameter was positively correlated with anxiety [all TS: *r*(16) = 0.57, *p* = 0.02; unmedicated TS: *r*(11) = 0.77, *p* = 0.01; Controls: *r*(11) = 0.45; *p* = 0.16]. Numerically, this relationship was strongest for unmedicated patients. Thus, whereas blink rate differentiated TS patients from controls, resting pupil diameter distinguished among TS patients with respect to severity of comorbid anxiety.

## Discussion

### Overview

Behavioral analysis of the Nemo task data revealed that YGTSS tic severity was strongly correlated with lower accuracy and slower RTs on the Mixed blocks of the Nemo task, even after controlling for ADHD or OCD symptom severity. This relationship, which was observed within the unmedicated sample as well as across the entire sample, replicates our prior findings with an independent sample of unmedicated TS patients who performed Mixed Nemo blocks (11). Indeed, TS patients showed a larger performance decrement than controls for the Congruency manipulation embedded in these challenging blocks. Thus, the Mixed-block variant of the Nemo task appears to be exquisitely sensitive to the cognitive difficulties experienced by children with TS.

Using temporally precise eyetracking methodology, we were able to confirm prior reports that patients with TS blink more than controls (18, 19). Indeed, patients exhibited an elevated number of short gaps within the range previously reported for spontaneous eyeblinks (31, 33). This group difference was observed both during cognitive control task performance and during a 30-s resting baseline period. Further, this difference was present both for unmedicated patients and for all patients relative to controls. By contrast, we found no evidence of a difference between TS patients and controls in average pupil diameter, and pupil diameter was uncorrelated with tic severity. Rather, pupil diameter correlated positively with MASC anxiety scores.

### Cognitive control in tourette syndrome

Some studies have reported cognitive control deficits in TS (10, 20), whereas others have reported the opposite (34, 35). These studies also vary as to whether they excluded medicated patients, factored in comorbidity symptom severity, and studied children with a recent diagnosis or adults who have learned to their tics over time. In fact, previous work from our lab showed a high degree of variability in cognitive control even within a sample of unmedicated children recently diagnosed with TS (11). Consistent with these prior findings, patients as a group did not perform worse than controls in the present study. However, tic severity among patients was strongly correlated with performance on the Nemo task. Indeed, performance on the Nemo task accounted for *half* of the variance on parental reports of tic severity. As such, this may well be the cognitive task that, to date, best captures the cognitive deficits commonly observed in TS. Given that tic suppression engages, broadly speaking, the same neural circuitry that underlies cognitive control (36), TS patients with better cognitive control would likely be better able to suppress their tics, thereby exhibiting lower tic severity scores on the YGTSS than they would otherwise.

An intriguing and heartening aspect of TS is that symptoms often, but not always, lessen as patients enter adulthood (37, 38). Several studies have found evidence of compensatory mechanisms in adults with TS, such as enhanced activation of PFC in TS patients relative to healthy adults, despite similar performance levels (39, 40). Adults with TS have been shown to exhibit enhanced cognitive control, or the ability to control one’s thoughts and actions, possibly due to years of tic suppression (34). Indeed, children who have the ability to effectively suppress their tics during childhood may exhibit increased PFC growth and amelioration of symptoms later in life (41). Thus, we propose that performance on the Nemo task could be a good predictor of a TS patient’s long-term prognosis; longitudinal investigation will be required to test this hypothesis.

### Spontaneous eyeblink rate

Prior studies involving manual video coding or electromyography had reported elevated blink rates in patients with TS than controls (18, 19). However, these studies did not control for the possibility that their measure of spontaneous eye blinks was contaminated by eye movement tics. Thus, we used eyetracking technology to test for an increased frequency of short-duration blinks, of the same duration as those measured in controls. This prediction was supported, even after controlling for ADHD severity, which may be associated with altered patterns of blinking (42). This finding is consistent with the hypothesis that heightened DA transmission in the central nervous system is central to the etiology of TS (13, 15).

Our other key prediction regarding blinks – a positive relationship between YGTSS tic severity and blink rate – was not borne out. Among patients, those with higher tic severity tended to have marginally *lower* blink rates. Several key points bear mentioning here. First, we were unable to collect viable data from patients who exhibited severe tics during eyetracking; the resulting sampling bias and relatively low sample size is important to keep in mind when interpreting these initial results. Second, the heightened spontaneous eyeblink rate in TS patients observed here should not be interpreted as the expression of ocular tics, which are assessed by the YGTSS. Third, as noted above, observed tic severity depends strongly on cognitive control, given that patients often voluntarily suppress phonic and motor tics; in contrast, even patients with good cognitive control are unlikely to be able to voluntarily suppress eye blinks for long. Finally, and related to the previous point, blink rate cannot distinguish between elevated dopamine in the striatum and prefrontal cortex, which should have differential effects on behavior and cognition in individuals with TS. However, we believe that the *combination* of measures of tic severity, cognitive control task performance, and ocular measures, such as spontaneous blink rate, could provide complementary insights about a patient with TS.

### Pupil diameter

Pupil dilation and heightened arousal are two of many bodily changes that occur in parallel upon activation of “fight-or-flight” sympathetic nervous system activity due to the actions of NE – a neuromodulator produced by the LC in the brainstem – on the eyes, brain, and many other organs. Thus, pupil dilation (in the absence of a change in luminance, which independently affects pupil diameter) can be considered a peripheral indicator of noradrenergic activity in the brain. Pupil diameter at rest is closely correlated with tonic activity of LC neurons in both monkeys and humans (21, 22). Thus, a finding that patients with TS have altered resting pupil diameters relative to age-matched controls would be consistent with the hypothesis that tonic noradrenergic levels are altered in TS. However, the present results do not support this hypothesis. Pupil dilation that is time-locked to the performance of a cognitively demanding task, known as *task-evoked pupil dilation*, is believed to result from the transient release of NE from the LC (43). Thus, a finding that patients with TS have altered task-evoked pupil dilation relative to age-matched controls would be consistent with the hypothesis that *transient* NE release in response to a challenge is altered in TS. Here again, the present results do not support this hypothesis.

The fact that pupil diameter did not differ between patients and controls suggests that differences in NE levels may not be a central feature of TS. However, we found that pupil diameter, both at rest and during task performance, was positively correlated with self-reported anxiety on the MASC and marginally positively correlated with OCD severity. Other studies have shown that anxious adults individuals show a larger amplitude pupillary response to painful or emotionally salient stimuli (44, 45); here, we find that anxiety in children is associated exhibit elevated pupil diameter both at rest and during performance of a cognitive task with affectively neutral stimuli. Given that many patients with TS experience anxiety, these findings are relevant for the disorder and indicate that pupil diameter provides complementary insights to blink rate (see Supplementary Material). It would therefore be of interest to test for reductions in resting and/or task-based pupil diameter over the course of treatment for anxiety in TS patients.

### Limitations and future directions

Further research is needed to address several limitations of the current study. First, the patients recruited here had a primary diagnosis of TS, and comorbid conditions as reported by parents. Future studies should use a larger sample and include patients with primary diagnoses of OCD and ADHD, as well as tic disorders that do not meet the DSM diagnostic criteria for TS. Second, although all patients had a primary diagnosis of TS given by a clinician, patients were not further evaluated in this study by a trained clinician, and the key index of tic severity was a semi-structured interview (YGTSS) conducted by a trained research assistant. However, the present work lays the foundation for future research employing eyetracking in conjunction with formal clinical evaluation. Third, both unmedicated and medicated children were included in most analyses, except for those assessing relationships with tic severity. Additional research is needed to determine how medications commonly used in TS influence cognitive control and ocular measures. Fourth, we did not obtain an independent measure of tics, e.g., via video coding. Tics could be a source of distraction during task performance, thus affecting accuracy or RT. However, participants with large amounts of invalid eyetracking data (i.e., those who are most likely to have been expressing tics while performing the task) were excluded from analysis, and further, among those patients who were included, there was no relationship between percentage of invalid data and either accuracy or RT. Lastly, TS is a disorder that fluctuates over time, and thus, it will be necessary to collect data at multiple time points to examine whether and how these ocular measures vary over time as a function of current tic severity and treatment regimen. Future work is necessary to determine whether spontaneous blink rate fluctuates with tic severity within patients, and whether spontaneous blink rate is a stable marker of the disease that can be detected even in remission – and perhaps even in unaffected siblings.

## Conclusion

The present findings establish a foundation for future research exploring whether cognitive control and eyetracking measures could help physicians select between various treatment options for individuals with TS and/or to monitor patients over time. Performance on our test of cognitive control proved to be an effective predictor of tic severity among patients, even when controlling for ADHD, thereby highlighting the fact that TS affects both cognitive and motoric functions. This finding is relevant for patients’ academic performance, given a prior study showing that better performance on this task is associated with better math and literacy skills in childhood (46). Blink rate was marginally negatively related to tic severity, contrary to expectations. Notably, however, elevated blink rate in patients relative to controls could be detected with as little as 30 s of eyetracking data collected at rest. Finally, pupil diameter, which was unrelated to tic severity, proved to be a potentially useful indicator of comorbid anxiety – one that could perhaps aid in detecting a predisposition toward anxiety before it becomes clinically apparent. Although the sample size in the present study did not enable a complete characterization of effects of medication status and comorbid conditions, these results set the stage for further research examining the possible clinical utility of the novel experimental paradigm described here. Longitudinal research will be needed to determine whether the ocular indices are trait-like or state-like, and whether they can be used to predict future clinical states.

## Conflict of Interest Statement

The authors declare that the research was conducted in the absence of any commercial or financial relationships that could be construed as a potential conflict of interest.

## Supplementary Material

The Supplementary Material for this article can be found online at http://journal.frontiersin.org/article/10.3389/fpsyt.2015.00095

## Footnotes

^1^MASC scores were available for only 11 of the controls. We therefore did not consider MASC scores as a covariate in the group comparisons.
