# Supplementary material for: Tourette Syndrome: Complementary Insights from Measures of Cognitive Control, Eyeblink Rate, and Pupil Diameter
Source: Front Psychiatry. 2015 Jun 29;6:95. doi: 10.3389/fpsyt.2015.00095 (PMC4484341; doi:10.3389/fpsyt.2015.00095)

Supplementary Material

I. Supplementary Methods

Clinical assessments

Child Behavior Checklist (CBCL).[1] All parents completed the CBCL, which includes subscales for internalizing and externalizing symptoms. We used the CBCL to screen our control participants, as well as to characterize behavioral problems in the patients. Reliability for this sample was good, Cronbach's alpha=.78.

Behavior Rating Inventory of Executive Function (BRIEF).[2] This parental report contains 86 items of eight empirically obtained clinical subscales that capture different aspects of executive function, including abilities to initiate and inhibit behaviors, select relevant task goals, plan and organize complex problems, and shift problem solving strategies flexibly. For this study, we focus on the overall Global Executive Composite score. Parents are asked to answer if their child has had a problem with the behaviors in each item in the previous six months on a three point Likert scale ranging from one = *never a problem* to three = *often a problem*. Within this sample, the scale had good reliability, Cronbach's alpha=.79.

Multidimensional Anxiety Scale for Children (MASC).[3] This well-validated 39-item self-report measure is given to children aged 8-19 years old. The MASC is designed to assess varying anxiety dimensions in children and adolescents, including physical symptoms ("I feel shaky or jittery"), harm avoidance ("I stay away from things that upset me"), social anxiety ("I have trouble asking other kids to play with me"), and separation/panic ("I try to stay near my mom or dad"). Children answered each item on a four point Likert scale ranging from zero to three (never true to often true). For this study, we focused on the total Anxiety Index score, which had excellent reliability, Cronbach's alpha=.90. Because this measure was added to the protocol at a later date, the MASC was administered to only 11 controls and 16 patients.

Yale Global Tic Severity Scale (YGTSS).[4] The YGTSS is a semi-structured interview given by a trained senior member of the research team. The interview was given with the child or adolescent

and the parent(s) together, and interviewer observations were also recorded. Because discussion of tics can actually increase the frequency and intensity, interviews were given upon completion of the behavioral tasks so that children wouldn't be as acutely aware of each of their tics. YGTSS measures the severity of both motor and phonic tics experienced by the child during the previous week (number of tics, frequency, interference, intensity, and complexity), as well as the level of impairment associated with these tics. Each of the severity questions is separately scored for motor and phonic tics on a five-point scale (for a maximum severity total of 50), while the impairment scale is scored by ten point anchors ranging from 0 to 50. Because the impairment score does not separate impairment due to TS or other comorbid disorders, our analyses were limited to consideration of the tic severity score. Due to a lack of clinicians during sessions, only one trained team member was available to administer interviews, thus removing the possibility of conducting reliability analyses.

Children's Yale-Brown Obsessive Compulsive Scale (CY-BOCS).[5] This semi-structured interview was administered by a trained senior member of the research team with input from the patient and parent(s). The CYBOCS is designed to assess the presence and severity of obsessive and compulsive symptoms in children during the previous week. The interview began with the interviewer reviewing definitions of obsessions and compulsions, followed by a detailed questioning. The four most severe obsessions and compulsions are selected and assessed for severity; ratings are the interviewer's best estimate based on child and parent response. The total score includes the sum of obsession severity, compulsion severity, and overall impairment.

Conners' ADHD Parent Rating Scale.[6] This is a well-validated, 28-item scale based on childhood and adolescent psychopathology and problem behavior. Parents are asked to respond to items based on their children's behavior in the past month and choose one response on a four point Likert scale, ranging from zero = *not true at all* to three = *very much true*. Within the rating scale, there are three subscales to capture a broad range of problem behaviors, including conduct ("Spiteful or vindictive"), cognition ("Forgets things he/she has already learned"), and social problems ("Disturbs other children"). For this study, we focused on the total ADHD Index score, which had good reliability within this sample, Cronbach's alpha=.82.

II. Supplementary Results

Behavioral results on the Nemo task

To test for effects of block type on performance, we ran Group x Block (Direction, Color, Mixed rule block) ANOVAs for Incongruent trial accuracy and response times (RTs). For accuracy, there was a significant main effect of Block ($F(2,64) = 28.16$, $p < .001$), driven by reduced accuracy in Color blocks relative to Direction and Mixed blocks (*p*s < .001; Supplementary Fig. 1A). There was no main effect of Group, nor was there an interaction between Block and Group (*p*s > .2); indeed, the effect of Block was present in both controls ($p < .001$) and in TS patients (All TS: $p < .001$, Unmed TS: $p = .024$). For RTs, there was also a significant main effect of Block ($F(2,64) = 33.19$, $p < .001$; Supplementary Fig. 1B). This effect was driven by increased RTs for Color versus Direction blocks ($p = .002$), and for Mixed versus both Direction and Color blocks (*p*'s < .001). There was no effect of Group on RTs.

Testing for a relationship between spontaneous eyeblink rate and pupil diameter

Individuals with high levels of circulating DA in the central nervous system might also be expected to have high levels of circulating NE, given that DA can be readily converted into NE. If so, one should predict, by extension, a positive correlation between spontaneous eyeblink rate (linked to dopamine levels) and task-evoked pupil dilation (mediated by NE). As we are not aware of any prior research correlating these two ocular measures, we tested this prediction using the Nemo task ocular data. Indeed, we found across all participants that individuals with higher blink rates exhibited larger pupil diameters on the Nemo task ($r(40) = .43$, $p = .005$). This relationship was significant in controls ($r(21) = .45$, $p = .04$) and marginal for all TS patients ($r(19) = .42$, $p = .07$), but was not significant for unmedicated patients alone ($r(13) = .27$, $p >$ .20). This relationship between baseline blink rate and pupil diameter measures also held when computed across all participants ($r(40) = .35$, $p = .028$). Moreover, despite this correlation between ocular measures, and the group differences that were observed for blink rate, we did not observe any effect of group on pupil diameter, either during task performance or during baseline

fixation (all ps > .20). Thus, although there was a positive relationship across all participants between eyeblink rate and pupil diameter, they are not redundant measures.

Testing the sensitivity of ocular measures to cognitive control task demands

Given that catecholamine levels are known to rise during cognitive challenges, we tested whether blink rate and average pupil diameter would increase across Direction, Color, and Mixed rule blocks. Additionally, we tested whether patients and controls responded differentially to this manipulation of task difficulty. As noted above, TS patients readily learned the rules of the Nemo task and implemented them accurately on the single-rule blocks, but had difficulty when the two task rules were intermixed. Thus, we sought to test whether TS patients would exhibit either heightened or blunted sensitivity to task difficulty, as measured via eyetracking.

First, we tested whether blink rate was influenced by task demands in patients and/or controls. To this end, we tested the effects of Block type on blink rate for all the patients and controls who completed all three block types. Supplementary Fig. 2A shows average blink rates for each block. In addition to the main effect of Group, described previously, a Group x Block type ANOVA revealed a significant main effect of Block type, with higher blink rates on mixed versus direction blocks ($p = .007$) and marginally higher blink-rates on color versus direction blocks ($p = .09$). Although the Group x Block type interaction was not significant ($p > .20$), it may be worth noting that the effect of Block type was highly significant in controls ($F(2,28) = 6.43$, $p = .005$) but was not significant in either unmedicated or all patients (p's > .20). Moreover, when each Block type was considered separately, patients demonstrated significantly higher blink rates compared to controls on both Direction ($t(32) = 3.02$, $p = .005$) and Color ($t(32) = 2.24$, $p = .03$) blocks, but only marginally higher blink rates on Mixed blocks ($t(32) = 1.79$, $p = .09$). Thus, TS patients exhibited elevated blink rates even on the easier task blocks.

Second, we tested whether pupil diameter was differentially influenced by task demands in patients versus controls. To this end, we tested the effects of Block type (Direction, Color, or Mixed) on pupil dilation in all the patients and controls who completed all three block types (Supplementary Fig. 2B). A Group x Block type ANOVA revealed only a trend-level main effect

of Block type ($F(2,64) = 2.17$, $p = .12$), with no significant main effect of Group ($p > .20$) or Group x Block type interaction ($p > .20$). The trend-level effect of Block type became non-significant when controlling for MASC scores. Confirming our initial analyses focused on the Mixed blocks, these analyses involving all three block types indicate that pupil diameter does not distinguish between patients with and without TS.

III. Supplementary Information about Patients

Subject	Age	Gender	Medications	Co-morbidities	Connor's ADHD index	CY-BOCS	YGTSS severity	Current Motor and Phonic tics
1+	8	F	none	none	42	6.50	14	eye blink, chin to chest then tilt head back (twice in a row), curling toes under, arm movement, humming, groaning
2*+	13	M	none	None	61	0	12	open mouth really widely, spitting (but if no saliva will still make the spitting noise), coprolalia
3*+	13	F	none		43	0	8	eye blink, nose scrunch, hand movement
4*+	10	M	none	OCD	45	8	14	flick eyes around, shoulder shrugs, shift hat/clothes around (especially sweatshirts), throat clearing
5+	7	M	none	none	64	6.50	24	eye blink, grab at shirt and pull off chest, fart noise with mouth, sighing, repeating words
6+	8	M	none	none	60	0	8	throat clearing
7*+	12	M	none	ADHD	67	0	9	eye blink, head twitch forward, hand/wrist twitch
8*+	13	M	none	none	41	3	9	eye blink, nose twitch
9*+	7	F	none	ADHD	89	0	12	twirling, skipping, cherry noise
10*+	10	F	none	ADHD, Generalized Anxiety Disorder	90	10	25	eye blink, facial grimace, knuckle cracking, kick shoe on ground, walk 3 steps and then hop on 1 foot then walk 3 steps hop on the other foot, coughing, throat clearing
11*+	8	M	none	none	52	10	22	eye blink, head/neck thrust, jumping, hair twirling, throat clearing, reverse of throat clearing, sniffing,
12*+	12	F	none	none	69	30.50	15	eye blink, eye widening, stomach/neck tensing, rub foot to back of leg while walking, coughing
13*+	12	F	none	ADHD, adjustment disorder	72	0	26	eye blink, eye roll, facial grimace with gasp, snapping fingers, blowing air out of nose

14[*+]	9	M	none	none	60	0	22	eye blink, head nod/shake, looking at sun, throat clearing, lip smacking
15[*+]	10	F	none	none	42	0	5	eye blink
16[*+]	9	F	none	OCD, ADHD	69	15	22	tensing lungs, "shiver" (brings arms forward and contorts face), breathing in deeply like a gasp, while reading changes breathing pattern
17[+]	10	M	none	none	47	0	20	eye blink, eye roll, shake/nod head, slapping fingers together, mouse squeak noise
18[+]	8	M	none	ADHD	83	0	20	eye blink, looking up with eyes, head move forward while talking, looking at palms, "he-he" noise
19[*+]	11	M	1mg Halodol, 2mg Risperidone, 50mg Sertraline, 300mg Oxcarbazepine	OCD, ADHD, Oppositional defiance	50	0	29	eye blink, face scrunch, hand movement, clenching arms, clapping, hitting things (sometimes self-abusively), grunting, saying sorry
20[*+]	12	M	50mg Stratera, 50mg Zoloft	OCD, ADHD	57	21	27	eye twitch, nose crunch, facial grimace+foot+hand jerk, cracking knuckles and neck, sneeze/cough, scream, "boop" noise
21[*+]	9	M	10mg Focalin, 2mg Tenex	ADHD	52	0	9	eye blink, eye roll, sniffling
22[*+]	13	F	20mg Dexameth, 3600mg NAC,	OCD, ADHD, Generalized Anxiety Disorder	74	0	10	coughing, burping, throat clearing
23	12	M	2mg Imipramine, 25mg Clorapramine	OCD, Generalized Anxiety Disorder	64	9	25	eye blink (rapidly), eye wandering, holding eyes with hands, head jerk, tensing abdomen so ribs go over hips, leg jerk, snorting, saying sexual and swear words
24[+]	12	F	50mg Zoloft, 20mg Aderall	OCD, ADHD, Generalized Anxiety Disorder	59	19	0	none present
25[+]	9	M	5mg Abilify, 1mg Klonadine	none	67	8	20	wrist twist, knee bend and cracking especially while walking, neck twitch, hair pulling, throat clearing
26[*+]	12	M	15mg Deplin, 150mg Zoloft, 7.5mg Abilify, NAC	OCD, ADHD, Generalized Anxiety Disorder	58	18	24	eye roll, leg bouncing, shoulder shaking, hold breath then push it out forcefully until squeaks, snorting, spitting noise

Note: * eyetracking data presented [+] behavioral data presented

References

1. Achenbach TM, Howell CT, Quay HC, Conners CK. National survey of problems and competencies among four- to sixteen-year-olds: Parents' reports for normative and clinical samples. *Monogr Soc Res Child Dev.* 1991;56(3):1-131.
2. Gioia GA, Isquith PK, Guy SC, Kenworthy L. Behavior rating inventory of executive function. *Child Neuropsychology.* Sep 2000;6(3):235-238.
3. March JS, Parker JD, Sullivan K, Stallings P, Conners CK. The Multidimensional Anxiety Scale for Children (MASC): Factor structure, reliability, and validity. *Journal of the American Academy of Child and Adolescent Psychiatry.* Apr 1997;36(4):554-565.
4. Leckman JF, Riddle MA, Hardin MT, et al. The Yale Global Tic Severity Scale: Initial testing of a clinician-rated scale of tic severity. *Journal of the American Academy of Child and Adolescent Psychiatry.* 1989;28(4):566-573.
5. Scahill L, Riddle MA, McSwiggin-Hardin M, et al. Children's Yale-Brown Obsessive Compulsive Scale: Reliability and validity. *Journal of the American Academy of Child and Adolescent Psychiatry.* Jun 1997;36(6):844-852.
6. Conners CK. Rating scales in attention-deficit/hyperactivity disorder: Use in assessment and treatment monitoring. *Journal of Clinical Psychiatry.* 1998;59 Suppl 7:24-30.

Supplementary Figure 1. A) Incongruent trial accuracy by block type in controls, unmedicated TS patients, and all TS patients. B) Incongruent trial response times by block type for each group. Asterisks denote $p < .05$.

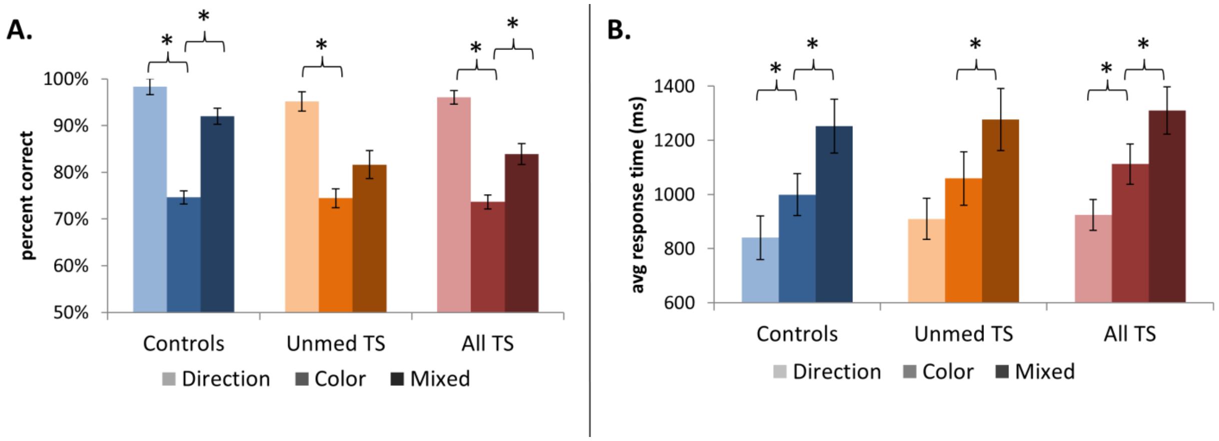

Supplementary Figure 2. A) Blink rate by block type (Direction, Color, or Mixed), for controls, unmedicated TS patients, and all TS patients. B) Average pupil diameter by block type. Error bars show within-subjects standard error. Asterisks indicate $p < .05$.

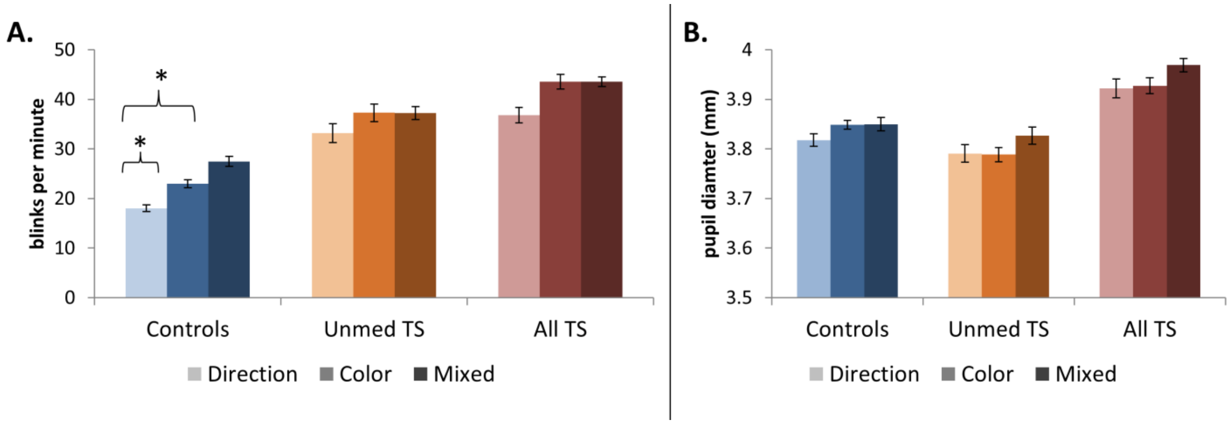

Supplement: Supplementary file 1 [file Presentation_1.PDF]
